# Prediction of atrial fibrillation from at-home single-lead ECG signals without arrhythmias

**DOI:** 10.1038/s41746-023-00966-w

**Published:** 2023-12-12

**Authors:** Matteo Gadaleta, Patrick Harrington, Eric Barnhill, Evangelos Hytopoulos, Mintu P. Turakhia, Steven R. Steinhubl, Giorgio Quer

**Affiliations:** 1 Scripps Research Translational Institute, La Jolla, CA USA; 2 iRhythm Technologies, San Francisco, CA USA; 3 Stanford University School of Medicine, Stanford, CA USA; 4 Purdue University, Weldon School of Biomedical Engineering, West Lafayette, IN USA

**Keywords:** Predictive markers, Risk factors, Atrial fibrillation

## Abstract

Early identification of atrial fibrillation (AF) can reduce the risk of stroke, heart failure, and other serious cardiovascular outcomes. However, paroxysmal AF may not be detected even after a two-week continuous monitoring period. We developed a model to quantify the risk of near-term AF in a two-week period, based on AF-free ECG intervals of up to 24 h from 459,889 patch-based ambulatory single-lead ECG (modified lead II) recordings of up to 14 days. A deep learning model was used to integrate ECG morphology data with demographic and heart rhythm features toward AF prediction. Observing a 1-day AF-free ECG recording, the model with deep learning features produced the most accurate prediction of near-term AF with an area under the curve AUC = 0.80 (95% confidence interval, CI = 0.79–0.81), significantly improving discrimination compared to demographic metrics alone (AUC 0.67; CI = 0.66–0.68). Our model was able to predict incident AF over a two-week time frame with high discrimination, based on AF-free single-lead ECG recordings of various lengths. Application of the model may enable a digital strategy for improving diagnostic capture of AF by risk stratifying individuals with AF-negative ambulatory monitoring for prolonged or recurrent monitoring, potentially leading to more rapid initiation of treatment.

## Introduction

Atrial fibrillation (AF) is the world’s most common sustained arrythmia, associated with a 5-fold increased risk of stroke^1–3^, but remains undiagnosed in about 10–20% of affected individuals^4,5^. Once identified, the likelihood of stroke for AF patients can be reduced with treatments such as oral anticoagulants^6^. However, despite a lifetime risk of developing AF of almost 25% for adults over age 40, the evidence of overall benefit for systematic screening relative to opportunistic in-clinic screening has been limited to date, leading to conflicting recommendations^7–9^. To alleviate the cost of non-targeted screening, risk stratification of individuals most likely to benefit from long-term monitoring would be of great clinical benefit^10^. One method of risk stratification can be enabled by future AF prediction from findings in a non-AF ECG signal, as shown with 10-s, in-clinic 12-lead ECGs in retrospective^11–13^ and prospective^14^ trials, or using single-lead 24-hour, non-AF ECG recordings to predict AF in the next 14 days^15^.

Predicting the risk of AF presence from an ECG signal that does not contain AF would allow the identification of the fraction of the population that may have paroxysmal AF but that was not detected with the initial ECG. Identification of increased risk could then enable a more targeted screening strategy to identify individuals who can benefit the most from prolonged ECG monitoring.

**Fig. 1:**
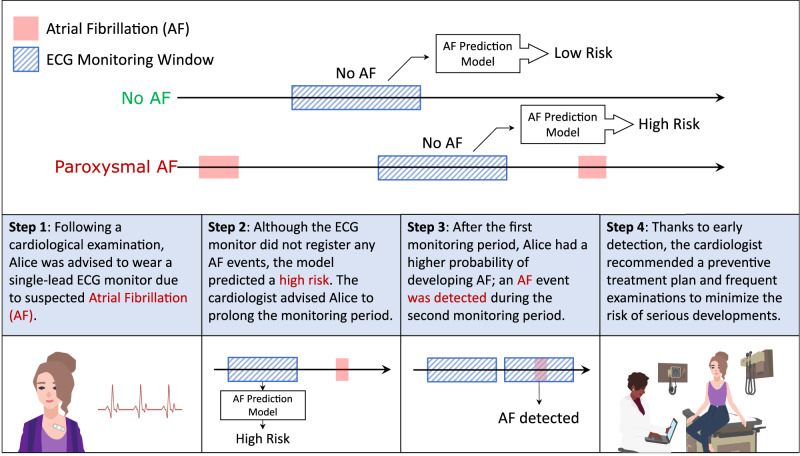
An example of application of the AF prediction model. The figure illustrates the possible outcomes of the AF prediction model when AF is not detected: it can predict low or high risk for future AF development. In the second part of the figure, we present an example of a potential application. Alice was advised to wear an ECG patch to monitor potential AF, and although no AF was detected, the model predicted a high risk of AF. Consequently, she was advised to wear a second ECG patch, which ultimately detected AF, allowing her to discuss appropriate treatment with her clinician.

The goal of the current work is to develop a model to identify near-term AF in a two-week window based on findings in non-AF single-lead ECG intervals of lengths from 10 min to 24 h. (Fig. 1) We found that a single-lead ECG signal significantly improves the prediction of future AF occurrence relative to the model not including ECG signal data.

## Results

**Fig. 2:**
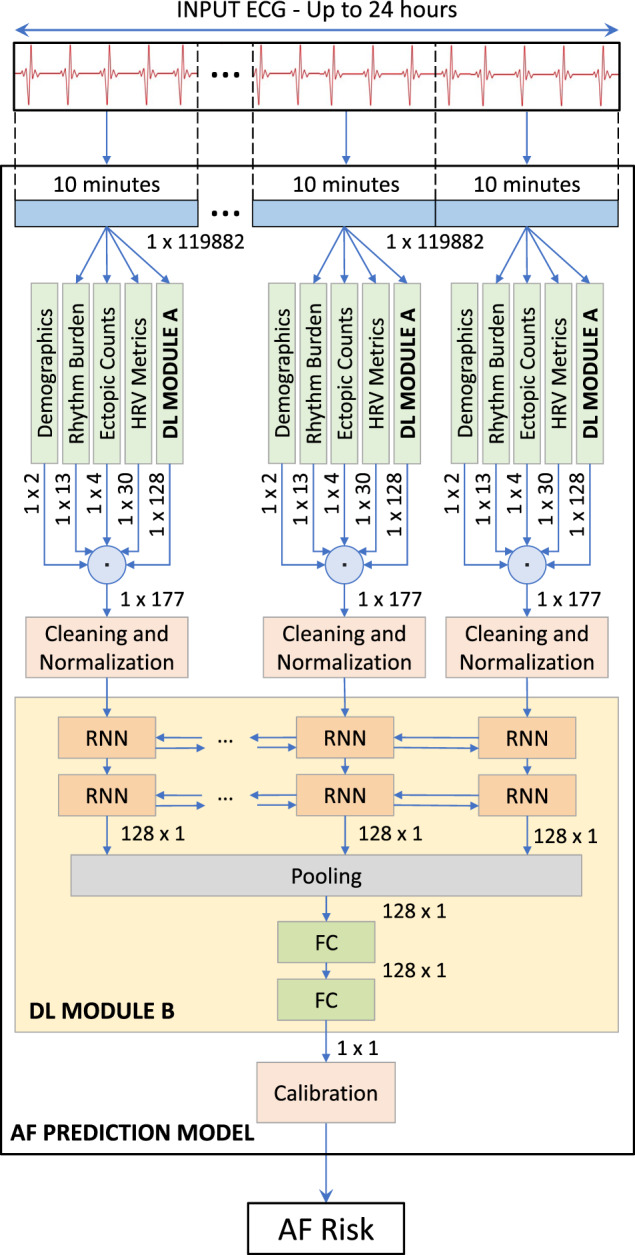
AF prediction model. The figure shows the processing pipeline from the raw input ECG to the final AF risk assessment. First, the ECG is divided into 10 min windows. Then, demographics, HRV, ectopic and abnormal rhythm, and DL features from DL module A are extracted from each of these windows. After cleaning and normalization, DL module B assesses the risk score of the entire input sample, which is adjusted by the calibration function.

Using 459,889 single-lead ECG recordings of length up to two weeks, we developed a model to estimate the risk of presenting AF in a two-week observation period by analyzing only non-AF ECG intervals of various length. In this context, AF risk is defined as the predicted probability of the occurrence of an AF event, as informed by the ECG data. The model works with different inputs, adaptable to different scenarios, while its output is always the risk of presenting AF (Fig. 2).

We refer to the same model with different names depending on the specific input considered. In particular, we consider the model with input age and gender (AG model), demographic and HRV features (AG + HRV model), deep learning features automatically extracted (DL Only model), manually extracted features excluding deep learning features (AG + HRV+Ectopic+Rhythm model), and all features including deep learning ones (All Features model; Table 1).

**Table 1:** Inputs considered by the model for AF prediction.

Model name	Model inputs
AG model	Demographics only – age and gender
AG + HRV model	Demographics and HRV features
AG + Ectopic model	Demographics and ectopic beat counts
AG + HRV + Ectopic + Rhythm model	Demographics, HRV features, ectopic beat counts and other manually extracted features
DL Only model	Only features extracted from the ECG by the deep learning network
All Features model	All features available, including both manually extracted and deep learning features

Naming of all variants of the proposed AF prediction model based on the choice of input available, among demographic characteristics (age and gender), HRV features, frequency of ectopic beats, other rhythm related features, and automatically extracted deep learning features.

### AF prediction performance for all individuals

When including all individuals in the cohort (AF prevalence 4.3%), the AUC was 0.67 (confidence interval, CI: 0.66–0.68) for the AG model; 0.74 (CI: 0.72–0.75) for the AG + HRV model; and 0.80 (CI: 0.79–0.81) for the All Features model. The *p*-value was <0.01 when comparing the performance of the AG model with the AG + HRV model, and <0.01 when comparing the AG + HRV model with the All Features model. With respect to the AG model, the All Features model shows an improvement in AUC of 0.12 (CI: 0.11–0.14), with a *p*-value < 0.01. Specificity, precision (PPV), and F1 score evaluated at a sensitivity of 0.80 are reported (Table 2).

**Table 2:** AF prediction performance at a given sensitivity of 0.80.

Feature set	Age group	Input ECG length	Tot. recordings	Prevalence (%)	Sensitivity	Specificity	Precision	F1 score
AG	<65	1 day	11,820	2.2	0.80	0.58	0.04	0.08
AG	≥65	1 day	11,329	6.5	0.80	0.26	0.07	0.13
AG	Overall	1 day	23,149	4.3	0.80	0.49	0.07	0.12
AG + HRV	<65	1 day	11,820	2.2	0.80	0.66	0.05	0.09
AG + HRV	≥65	1 day	11,329	6.5	0.80	0.37	0.08	0.15
AG + HRV	Overall	1 day	23,149	4.3	0.80	0.56	0.08	0.14
DL Only	<65	1 day	11,820	2.2	0.80	0.72	0.06	0.11
DL Only	≥65	1 day	11,329	6.5	0.80	0.51	0.10	0.18
DL Only	Overall	1 day	23,149	4.3	0.80	0.65	0.09	0.17
All Features	<65	1 day	11,820	2.2	0.80	0.76	0.07	0.13
All Features	≥65	1 day	11,329	6.5	0.80	0.50	0.10	0.18
All Features	Overall	1 day	23,149	4.3	0.80	0.65	0.09	0.17

Specificity, precision, and F1 score evaluated at a given sensitivity of 0.80 when considering a 1-day single-lead ECG as input, for people under the age of 65, for people above 65, and overall. Prevalence and total number of recordings for each group are also reported. The performance is reported for the model with four different input configurations: age and gender (AG); age, gender, and HRV (AG + HRV); deep learning features only (DL Only); all input available (All Features).

**Fig. 3:**
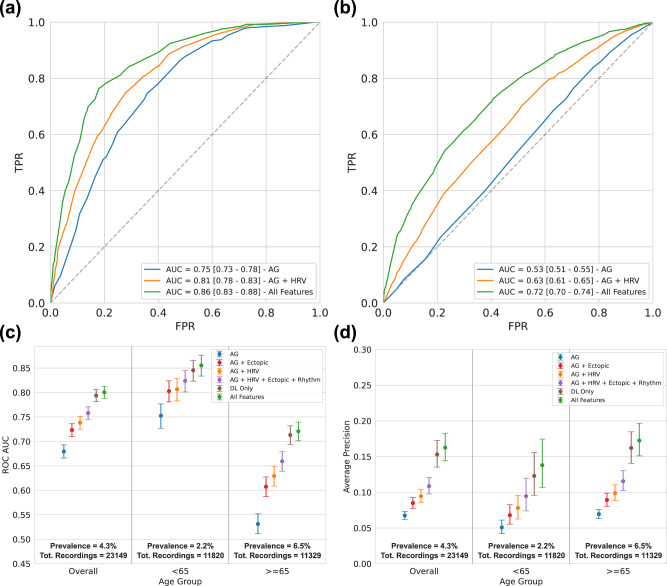
AF prediction performance per Age. Receiver operating characteristic (ROC) curves of the model when considering a 1-day single-lead ECG as input, for people under the age of 65 (**a**), and for people above 65 (**b**). The overall (and age specific) area under the ROC curve (AUC) (**c**) and Average Precision (**d**) are reported along with prevalence and total number of recordings for each group. The performance is reported for the model with six different input configurations: age and gender (AG, blue lines); age, gender and HRV (AG + HRV, orange lines); age, gender and frequency of ectopic beats (AG + Ectopic, red lines); age, gender, HRV, ectopic beats and presence of other rhythms (AG + HRV + Ectopic + Rhythm, purple lines); deep learning features only (DL Only, brown lines); all input available (All Features, green lines). The uncertainty bars represent the 95% confidence interval (CI) evaluated with a 10,000 iterations boostrap method.

The performance of the DL Only model, which received input of deep learning features derived from the ECG, was superior to the performance of the model receiving any input but deep learning features (AG + HRV +Ectopic + Rhythm). The DL Only model performed almost on par with the All Features model, with a minimal difference in AUC (CI: 0.00–0.01, *p*-value = 0.02; Fig. 3).

### AF prediction performance stratified by age

The probability of having at least one AF episode during two weeks of monitoring was strongly dependent on the age of the individual, with prevalence of 2.2% and 6.5% for individuals younger or older than 65 years old, respectively. The area under the curve (AUC) of the receiver operating characteristic curve (ROC) for the All Features model was 0.86 (CI: 0.83–0.88) for those younger than 65 years and 0.72 (CI: 0.70–0.74) for those older than 65 years old. In comparison, the AUC for the AG model was 0.75 (CI: 0.73–0.78) and 0.53 (CI: 0.51–0.55) for younger or older than 65 years old (Fig. 3).

### AF prediction performance based on monitoring length

**Fig. 4:**
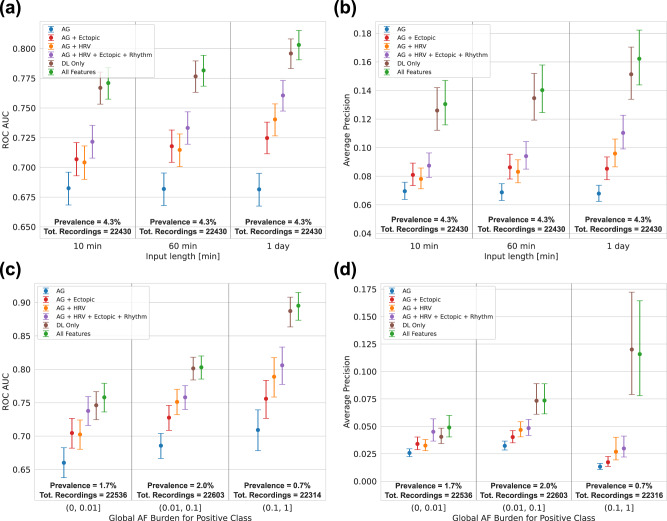
AF prediction performance per ECG length and AF burden. AUC (**a**, **c**) and Average Precision (**b**, **d**) of the model for different lengths of the input ECG (**a**, **b**), and for different AF burden ranges (**c**, **d**). Prevalence and total number of recordings for each group are also reported. The performance is reported for the model with six different input configurations: age and gender (AG, blue lines); age, gender and HRV (AG + HRV, orange lines); age, gender, and frequency of ectopic beats (AG + Ectopic, red lines); age, gender, HRV, ectopic beats and presence of other rhythms (AG + HRV + Ectopic + Rhythm, purple lines); deep learning features only (DL Only, brown lines); all input available (All Features, green lines). The uncertainty bars represent the 95% CI evaluated with a 10,000 iterations boostrap method.

A longer monitoring window used as input provided a more accurate prediction. Increasing the length of the single-lead ECG signal window used as input had a significant positive effect on its accuracy, both in terms of AUC and average precision. The All Features model attained an AUC of 0.77 (CI: 0.76–0.78) and 0.80 (CI: 0.79–0.81), *p*-value < 0.01, for acquisition lengths of 10 min and 24 h, respectively. The corresponding average precisions were 0.13 (CI: 0.12–0.15) and 0.16 (CI: 0.14–0.18), *p*-value < 0.01. As expected, the AG model was not affected by the acquisition length (Fig. 4).

### AF prediction performance based on AF burden

For each individual with AF, the AF burden was estimated using the entire acquisition period, and the population divided into 3 ranges: low (<1%), medium (from 1 to 10%), and high (>10%) AF burden. The AUC for the All Features model improved with increasing burden; 0.76 (CI: 0.74–0.78), 0.80 (CI: 0.78–0.81), and 0.90 (CI: 0.87–0.92) for individuals with low, medium, and high AF burden. In comparison, the AG model obtained an AUC of 0.66 (CI: 0.64–0.68, *p*-value < 0.01), 0.69 (CI: 0.67–0.70, *p*-value < 0.01), and 0.71 (CI: 0.68–0.74, *p*-value < 0.01) for individuals with low, medium, and high AF burden. For each burden range, the use of DL features allowed for improved prediction. This was particularly relevant in the medium (1–10%) AF burden range, where the All Features model achieved an AUC (0.80) that was 0.11 higher with respect to the AUC of the AG model (0.69), suggesting an improvement in separation of disease status for patients with potentially clinically relevant AF events not easily detected during a 1-day monitoring session (Fig. 4).

## Discussion

Our results show that it is possible to predict the occurrence of AF by analyzing a single-lead ECG obtained from a chest patch that does not contain any AF. While known parameters, including demographic characteristics (age and gender), HRV metrics, and ectopic beat frequency contribute to that prediction, it was only with the inclusion of morphologic analysis of the single-lead ECG with a deep learning approach that it was possible to obtain the most accurate prediction. These results were achieved by developing a framework for incorporating deep learning features extracted from single-lead ECG signal, along with demographic and phenotypic variables to predict occurrence of AF. The contribution of each feature category was assessed by analyzing different input configurations of the model, where the corresponding features were either included or excluded. This framework can predict the presence of AF within a 14-day period by observing a short window, from 10-min to 1-day, without AF. The results showed that a longer monitoring period improves the prediction, supporting the hypothesis that some features related to the presence of AF may not always be present in the signal and can be observed only with a longer monitoring window.

This work builds on our earlier work on the detection of AF from single-lead ECG signals^16^ and on the explanation of the deep learning features used^17^. Screening for AF has been demonstrated in multiple studies to substantially improve the detection of silent AF^18–23^, with some evidence of a reduction in long-term adverse outcomes^22,24^. Several groups have developed models to predict the future clinical diagnosis of AF using 10-s 12-lead ECGs performed in the clinic^11,13,25^, but their performance in detecting individuals with asymptomatic and low-burdens of AF requires further clarification as these individuals are unlikely to be detected with short and intermittent ECG follow-up^26,27^. The value of AF prediction from a 12-lead ECG without AF occurrences has recently been shown in a prospective study, with a specificity of 98% but a sensitivity of only 7.5% in the proposed operating point^14^. A longer rhythm observation has been performed with Holter monitors, showing the advantage of considering an interval longer than the 10-s, 12-lead ECG in order to improve AF prediction, with an AUC, a sensitivity, and specificity of 0.79, 76%, and 69%^15^. While previous studies have explored AF prediction using short 12-lead ECGs, our study complements these investigations by analyzing a single-lead ECG in a scenario with an extended duration of monitoring, which allowed us to also detect individuals with low AF burden that a brief 10-s 12-lead ECG snapshot might miss. This emphasis on prolonged analysis offers a more comprehensive view of an individual’s AF profile, filling in the gaps left by shorter duration monitoring methods.

Throughout most of the 100+ year of ECGs being a standard diagnostic test, its use has been primarily limited to clinical setting. This is rapidly changing with multiple technologies enabling anyone, at any time, to obtain at least a short single lead ECG through their smartwatch or other smartphone connected device^28^. In addition, with over 20% of the US adult population already regularly using a smartwatch or activity tracker, and several large manufacturers already incorporating regulatory-approved irregular heart rhythm notifications based on photoplethysmography signals, a rapidly expanding proportion of the population will require ECG confirmation of concerning self-reported findings, which may take seconds, days, or weeks of ECG monitoring^29^. As these technologies become ever more ubiquitous, the diagnosis and management of cardiac arrhythmias will likely change substantially relative to current systems of care and will require more sophisticated tools to identify those who would benefit the most from longer-term ECG monitoring. Since AF is the most common sustained arrhythmia, although often not diagnosed until the occurrence of an irreversible event such as a stroke or new heart failure^30,31^, identifying those at highest risk for AF remains a large unmet need.

The clinical diagnosis of AF is often dependent on an in-clinic 10-s 12-lead ECG. While this is adequate for those with persistent AF, for those with paroxysmal AF, estimated to be ~one-third of the AF population, this brief snapshot of heart rhythm would be unlikely to overlap with an AF episode. The use of Holter monitors and ECG chest patches have opened the possibility of non-invasive longer monitoring, allowing for an increased likelihood of detection of paroxysmal AF that depends on the duration of monitoring^26^. Indeed, especially for individuals with a low AF burden, in case an AF episode was not detected during the monitoring period, the risk of underlying paroxysmal AF remains unknown. Recent work using 12-lead ECGs and this work, as well as a prior studying analyzing prolonged single-lead ECGs, have shown how it is possible to predict the occurrence of an AF event and quantify the risk, based on the observed ECG signal, for individuals that do not present AF.

In this work, we have demonstrated that while HRV metrics, ectopic and rhythm-related features can provide an important contribution towards the prediction of AF with respect to a simple age and gender model, the analysis of the ECG morphology improves this prediction. First, the identification of the presence of ectopic beats, in particular premature atrial contractions (PACs), provides a small but independent contribution to the prediction. Most importantly, it is only with an automated extraction of representation learning features from the single-lead ECG signal morphology with a deep learning approach that we can obtain the best performance. We should also note that the model with deep learning features alone is capable of prediction almost on par with the case in which the full set of features is provided as input, suggesting an effective extraction of representative characteristics of the ECG during the training phase of the model. Our model can complement standard detection techniques, allowing the selection of the few individuals at higher risk, that could benefit from a second monitoring period in case no AF is detected during the first monitoring period. This could potentially allow for a better use of limited resources for AF detection and overall improved sensitivity of screening programs.

This work reinforces the potential to accurately predict the presence of paroxysmal AF in individuals with no findings of AF on their ECG. While we proposed an approach able to integrate stable characteristics (demographic) with HRV metrics and a continuous single-lead ECG signal, it is likely that the best prediction performance will be obtained only by collecting all available information including genetic and electronic health record data of the individual^32^, and integrating this information in a truly multi-modal approach^33^.

In the interpretation of the results of this work, one should consider that the retrospective data used for training and testing of the model were obtained from individuals clinically prescribed to use a device for monitoring their heart rhythm for an extended period of time, thus they may not be representative of the general population. A future study with a prospective validation would be needed before extending the applicability of the prediction model to the general population. Our analysis is also based on the single-lead ECG acquired during a period of up to 14 days, as well as demographic characteristics of the individuals, while pre-existing conditions, comorbidities, treatments, diagnoses, and other relevant information about the individual are not available. Race and ethnicity were not available in the retrospective dataset used for this analysis, potentially limiting the generalizability of the presented results. Furthermore, the presence or absence of AF is subject to the duration of these recordings since the individual may exhibit AF events outside the monitoring window. Finally, although the current modeling framework is extendable to other sensors, the specific model might not be generalizable to other wearable devices, requiring specific calibration to different sensor characteristics.

These results show that our deep learning approach, integrating the morphologic analysis of the single-lead ECG in the absence of AF, significantly improved near-term AF prediction relative to models including all other available features. Such improved prediction may enable a digital strategy to test individuals and provide a risk score for future AF based on their single-lead ECG, enabling a more targeted approach for extended monitoring of individuals at increased risk.

## Methods

We developed a model that can estimate an individual’s risk of developing atrial fibrillation or atrial flutter within a two-week observation period. Atrial fibrillation and atrial flutter are grouped into a singular category (referred to as just AF further) due to their overlapping clinical characteristics, shared treatment strategies, and the frequent coexistence in patients, reflecting a common underlying atrial electrophysiological disruption. This model takes an interval of the single-lead ECG signal as input, with a length between 10 min and 24 h, that does not contain any instances of AF (Fig. 2).

### Ethical considerations

The protocol for this study received approval from the Scripps Office for the Protection of Research Subjects and was deemed exempt from formal committee review (IRB 21–7719). The requirement for written consent was waived due to the retrospective nature of the study, which utilizes de-identified data.

### Characteristics of the individuals included in this study

The individuals included in this study were prescribed the iRhythm Zio®XT patch (iRhythm Technologies Inc, San Francisco, CA) as part of routine care to monitor their heart rhythm for up to 14 days. It is an FDA cleared, single-use, water-resistant, continuous ambulatory single-lead ECG skin adhesive patch (Supplementary Fig. 1).

For the development of the proposed model, we used the raw ECG signal directly acquired by the device, as well as the annotation in the clinical report, including arrhythmia detection, presence of ectopic beats, and estimated heart rate. The outcome of the study is defined as the presence of an AF event, which is an arrhythmia event that persisted for more than 30 s and was verified as AF by an iRhythm certified cardiographic technician. Individuals were categorized as AF positive if they exhibited at least one AF episode at any point during the 2-week single-lead ECG monitoring. We note that while the detected AF episode might be the initial recorded instance during the study, it is unlikely that it represents the patient’s very first episode with AF. Given the sporadic nature of AF, it is conceivable that individuals experienced unrecorded episodes prior to any observation.

No personal identifiable information (PII) was used in the extraction/selection of the data. We included recordings acquired from January 2019 to May 2022, excluding individuals with persistent or near-persistent AF (defined as AF burden over 70%). A total of 459,889 recordings, obtained from 446,900 unique individuals, were analyzed in this study. The recordings were divided into three different cohorts: (1) a training cohort, (2) a calibration cohort, and (3) a testing cohort.

To ensure accurate and reliable model performance across different age groups, we trained and validated a separate model for each subgroup based on age (ages 18–54, 55–64, 65–74, 75–84, and 85–99). The training cohort for each subgroup was enriched with additional recordings of AF to ensure that 50% of individuals with AF were detected. We accomplished this by randomly selecting 30,000 recordings for each age group, and then including up to an additional 30,000 recordings (or the total amount available if less than 30,000) that contained at least one AF event. As a result, the training cohort for each age group consisted of up to 60,000 recordings. The total number of recordings is 269,889, which is lower than 300,000 since for some groups less than 30,000 recording with at least one AF event were available. Of these recordings, 131,706 had underlying paroxysmal AF, with a median burden of 4.0% (inter-quartile range, IQR: 0.9–14.8%).

The calibration cohort was used to calibrate the AF prediction model. The calibration routine corrected for differences between the training cohort and an orthogonal cohort of data taken at a natural prevalence from a desired population. This corrected for factors like the higher number of AF detected cases present in the training. This correction puts the risk scores from the model trained for any age range on the same scale with intuitive interpretation, unifying the risk score across all patients. The calibration cohort contained 150,000 recordings, 30,000 for each age subgroup, from individuals not included in the training set (natural prevalence, without selection based on presence of AF). The testing cohort was used for evaluating the model performance and it was strictly separated from the other two cohorts. It contained 40,000 recordings selected at random after excluding the recordings from individuals included in the training or calibration cohorts (Supplementary Fig. 2).

### Model for the prediction of AF

The presented model was developed as a two-stage process for the analysis of the input ECG signal, combined in a hierarchical way. In the first stage, the model extracts several sets of features from 10 min of ECG data, as described in the following:

- first, a collection of standard heart-rate variability (HRV) metrics is evaluated utilizing the location of the R-peaks, excluding ectopic beats, which are considered separately;
- then, metrics related to other abnormal rhythms (e.g., supraventricular tachycardia, atrioventricular block) and ectopic beats are evaluated from the available annotations, including PACs and premature ventricular contractions (PVCs);
- finally, a deep learning module (DL module A) is used to automatically discover a set of representation learning features that provides a rich and compact representation of the single-lead ECG given as input.

Thanks to its modularity, each of these categories could be included or excluded from the final model. For comparison, we trained the model with different input configurations to show the benefit of including each one of these categories in the prediction. The full feature set and the corresponding category are reported (Supplementary Table 1).

In the second stage, the features extracted from multiple consecutive 10-min intervals were used as input for a second deep learning module (DL module B), which integrates all the available information collected over a longer period of time with demographic features. This hierarchical structure allows the model to focus on the local characteristics of the ECG during the first stage, while in the second stage the temporal relationships of the multi-dimensional signal are considered on a larger scale and leveraged to estimate the risk of future AF events (Fig. 2).

### Representation learning features

The proposed model aimed to enhance the accuracy of predicting AF from ECG data by leveraging long-term temporal relationships. However, recurrent neural networks (RNNs) face difficulties in processing long multi-dimensional sequences, limiting the number of short windows that can be used within a 1-day time frame. As a result, it was essential to identify a window size that provided enough temporal information to train a model effectively without making the sequence excessively long and complex. A 10-minute time frame has been empirically found to be a good compromise between these factors. It allows enough temporal information to be captured, while keeping the sequence short enough to be analyzed efficiently by the RNN. (Supplementary Material 1) DL module A was thus trained with the goal of extracting a set of representative features predictive of future AF events from an ECG period of 10 min without AF. For each 10-minute interval, the model analyzed and encoded the ECG data into a set of 128 outputs that contained all the essential information required for the following steps. These 128 features were taken from the second-last fully connected layer. (Supplementary Fig. 3) Once the DL module A was trained, its weights were considered frozen and no longer updated in the second phase (Fig. 2).

### Long ECG processing

A longer input signal (up to 1 day) was considered for the final assessment of the probability of AF occurrence. The set of features extracted during the first stage was computed for each 10-minute interval, and the resulting sequence was then processed by DL module B, which consisted of a 2-layer bidirectional long short-term memory (LSTM) neural network^34^, followed by 2 fully connected layers. During this optimization process, the pre-trained DL module A operated as a feature extractor, while DL module B was trained using an analogous process (Supplementary Material 2).

To map the model’s output to a real risk probability, we employed a calibration process. Specifically, we calibrated the model’s output scores for each age group using the calibration cohort, which followed a natural distribution. This allowed us to convert the model’s specific outputs to the probability of observing an AF event in the given monitoring window. Given the large size of each calibration cohort ( > 18,000 recordings), we utilized isotonic regression to calibrate the risk scores. The resulting output of the calibration step represents the final probability of developing AF. All the presented results refer to the calibrated outcome.

### Cleaning, processing, and normalization

The set of features extracted during the first stage from a 10-minute ECG included HRV, ectopic and abnormal rhythm, and DL features, along with age and gender. Since these features have different scales, ranges, and distributions, a processing pipeline was necessary to ensure that they could be effectively analyzed in the subsequent steps. The processing consisted of 3 parts: (1) data normalization or exponential transformation, (2) offset removal, and (3) scaling.

To handle the exponentially distributed variables, which were typical of rhythm burdens, we applied an exponential transformation of the form 1 - exp(-x), after removing the offset and scaling the values. For the remaining features, we utilized a Box-Cox transformation to achieve an approximately normal distribution^35^. All the parameters and the individual processing are reported (Supplementary Table 1).

ECG signals that were excessively noisy or contained a high number of artifacts were not included in the training dataset. Specifically, signals were considered invalid if any of the following conditions were met: 1) the average heartbeat was below 20 beats per minute, 2) a period longer than 2 min in the 10-minute window was automatically labeled as an artifact, 3) the number of detected R peaks was too few or too sparse to allow for the correct computation of the HRV metrics. These exclusion criteria helped to ensure the quality and reliability of the data used.

### Performance evaluation

To evaluate the predictive importance of the features considered, we trained and tested the model with six types of input configurations: demographic characteristics only (age and gender, AG); demographics and HRV (AG + HRV); demographics and frequency of ectopic beats (AG+Ectopic); demographics, HRV, ectopic beats, and presence of other non-sustained rhythms (AG + HRV + Ectopic + Rhythm); features from DL module A only (DL Only); and all available information (All Features; Table 1).

To assess how the acquisition length affected the AF prediction capabilities, we equalized the test cohorts by only considering samples with no AF events during a full day. We identified three potential scenarios of interest:

- a short monitoring time (10 min), a reasonably short signal acquisition time that may be acquired in a clinical setting or potentially at home;
- a medium monitoring time (1 h), that can be requested for patients needing additional screening and can potentially be performed as part of one clinical encounter;
- a longer monitoring time of 24 h, as is routinely carried out through the use of a wearable, portable device outside the clinical setting.

Finally, to assess the relationship between model performance and AF burden, the population was divided into three ranges: low (<1%), medium (from 1 to 10%), and high (>10%) AF burden. The performance of the model for each of these subgroups was based on a 1-day monitoring window.

To assess and compare the performance of all the presented scenarios, we utilized AUC and average precision metrics. We estimated confidence intervals using a bootstrap percentile-based method by repeatedly sampling the dataset with replacement for a total of 10,000 iterations, and we reported them with a confidence level of 95%. To compare different outcomes over the same population, we evaluated relative improvements and *p*-values using a paired bootstrap test, where the difference in performance was measured on identically sampled bootstrap iterations. For the estimation of *p*-values, we considered a two-sided paired test.

### Reporting summary

Further information on research design is available in the Nature Research Reporting Summary linked to this article.

### Supplementary information

Supplemental Material

Reporting Summary

## Data Availability

The data used in this study were obtained from iRhythm Technologies and are subject to restrictions that prevent public sharing, due to the terms of the data use agreement and compliance with ethical and legal requirements.

## Code availability

The code of the DL modules architecture is publicly available online: https://github.com/scripps-research/af-prediction.
