# Supplementary material for: Prediction of atrial fibrillation from at-home single-lead ECG signals without arrhythmias
Source: NPJ Digit Med. 2023 Dec 12;6:229. doi: 10.1038/s41746-023-00966-w (PMC10716265; doi:10.1038/s41746-023-00966-w)
Supplement: Supplementary file 1 — Supplemental Material [file 41746_2023_966_MOESM1_ESM.pdf]

SUPPLEMENTARY INFORMATION

Supplementary Material 1 - ECG representation learning (DL Module A)

The goal of DL Module A is to extract the best set of representative features associated to the occurrence of future AF events from a short 10-minute ECG record. We identified three main characteristics of the AF severity, which are measured on the entire acquisition (from 7 up to 14 days): 1) presence of AF, which is 1 if an event has been observed outside the short 10 minutes input ECG, otherwise 0 (y_1); 2) the AF burden, measured as the fraction of time in AF status divided by the full acquisition length (y_2); 3) the duration of the longest AF episode (y_3).

Due to the limited length of the acquisition, the uncertainty of the measured AF burden increases for lower burden values. To compensate for this, we define a corrected AF burden as the upper bound of the measured burden , as described in equation (1).

$$y_2 = measured\ burden\ +\ 0.1 * \frac{30}{acquisition\ length\ [seconds]} \quad (1)$$

Given a minimum clinically relevant AF event duration of 30 seconds, this adjustment will associate to individuals with no AF events recorded their worst-case burden, as if they have a short 30-second AF just after the end of the acquisition. In this way, the network's label for control cases (without AF) is very small but never exactly zero.

The network is modeled as a multiple regressor trying to infer these three metrics (y_1, y_2, and y_3), thus having a three-dimensional vector $\boldsymbol{o_A} = [o_1, o_2, o_3]$ as output. (Supplementary Figure 3)

An Adam optimizer with decoupled weight decay regularization[36] has been used to minimize the loss function, as presented in Equation (2).

$$
\begin{gathered}
loss_A(\boldsymbol{o_A}, \boldsymbol{y_A}) = l_1(o_1, y_1) + l_2(o_2, y_2) + l_3(o_3, y_3) \\
l_1(o_1, y_1) = -\Big(y_1 * log\big(\sigma(o_1)\big) + (1 - y_1) * log\big(1 - \sigma(x_1)\big)\Big) \\
l_2(o_2, y_2) = \big(o_2 - log(y_2)\big)^2 \\
l_3(o_3, y_3) = \big(o_3 - log(y_3 + 10^{-3})\big)^2
\end{gathered}
\qquad (2)
$$

Where $\boldsymbol{o_A} = [o_1, o_2, o_3]$ is the output vector of the final linear layer of the DL module A, $\boldsymbol{y_A} = [y_1, y_2, y_3]$ is the set of labels described above, and $\sigma(x)$ is the sigmoid function. Each of the output o_1, o_2, and o_3 of the model is associated with the corresponding label y_1, y_2, and y_3, representing the three main AF characteristics under analysis. The equation refers to a single sample. A batch of 32 different random samples have been used for each iteration, and the average loss was used for the optimizer update.

Supplementary Material 2 - Long-term data relationship (DL Module B)

DL Module B aims to estimate the uncalibrated AF risk score from longer (up to 1 day) recordings. The network is modeled as a binary classifier with the capability of providing probability estimates for the positive class.

Using data randomly sampled from the training cohort, the optimizer minimizes a binary cross-entropy loss function (Equation 3), which only depends on the presence of AF events outside the input period (y_B).

$$loss_B(o_B, y_B) = -\left(y_B * log\big(\sigma(o_B)\big) + (1 - y_B) * log\big(1 - \sigma(x_B)\big)\right) \tag{3}$$

Where o_B is the mono-dimensional output of the last linear layer of DL module B. Since the training cohort has been enriched by additional positive cases, and a different model has been trained for each age range, o_B cannot be interpreted as a direct estimation of the AF risk and a calibration process is necessary.

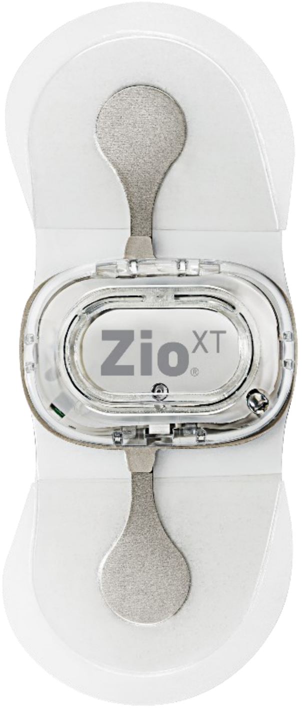

Supplementary Figure 1 – iRhythm ZioPatch XT.

The iRhythm ZioPatch XT, a small and wearable patch designed for monitoring and diagnosing cardiac arrhythmias, was used to acquire the data for this study. This medical device can continuously record a patient's single-lead electrocardiogram (ECG) for up to 14 days, making it a reliable tool for long-term monitoring. The device allows patients to continue their daily activities without interference. It has a water-resistant design, so patients can wear it while showering or exercising. The patch is applied to the patient's chest and is removed after the monitoring period is complete.

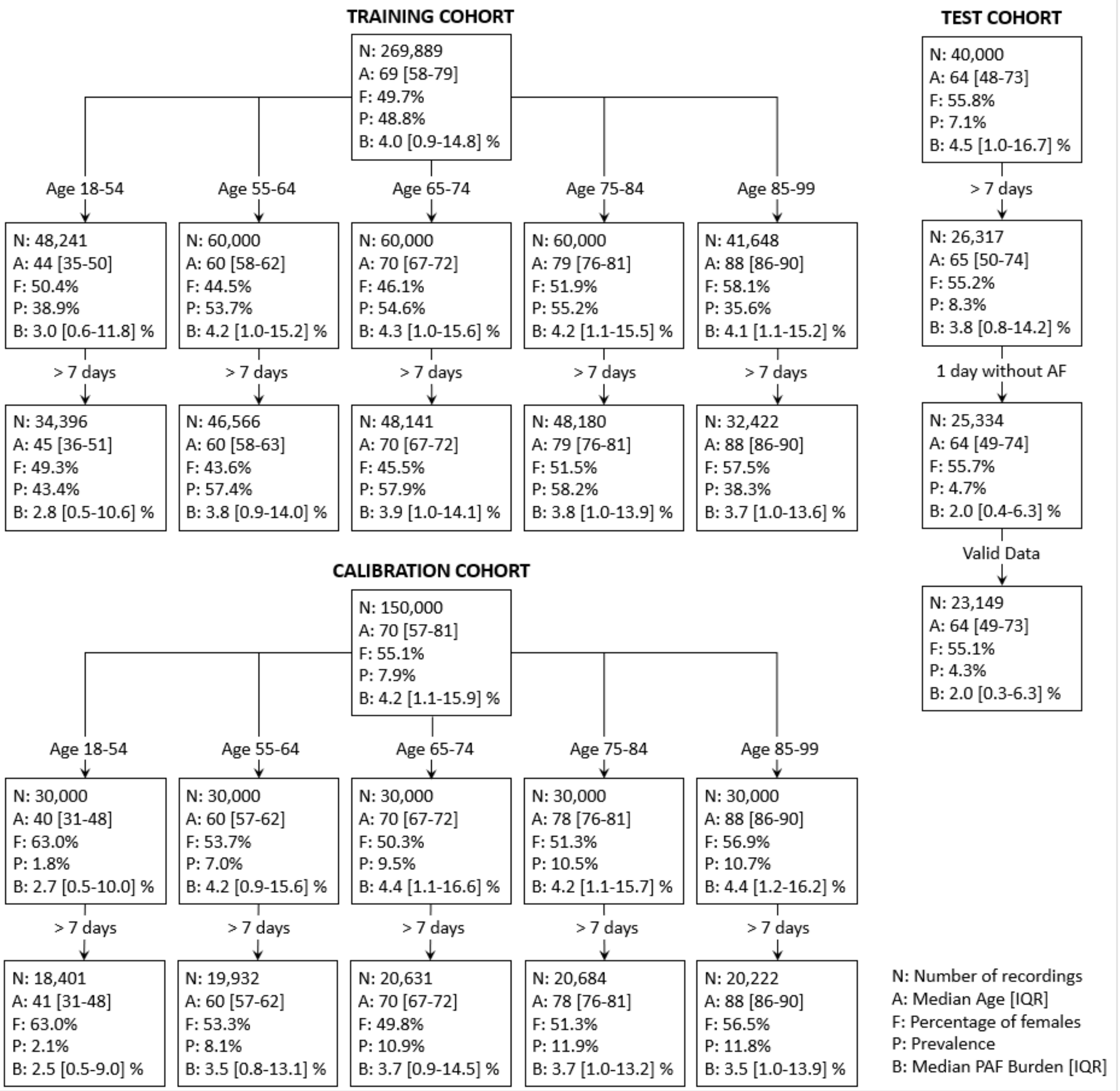

Supplementary Figure 2 – Cohort description.

Number of recordings (N), age (A), percentage of females (F), prevalence (P), and AF burden for PAF cases (B) are reported for each cohort, and after each selection step. Acquisition shorter than 7 days are excluded from the analysis. For the test cohort, random 1-day intervals are sampled, and only those with valid data and not presenting any AF are included in the testing phase.

Feature	*Category*	*Box-Cox Lambda*	*Offset*	*Scale*	*1 - exp(-x)*
Gender	*Demographics*	*NA*	*0.00*	*1.00*	*No*
Age	*Demographics*	*NA*	*0.00*	*100.00*	*No*
Mean_NN interval	*HRV*	*0.4438*	*42.86*	*5.69*	*No*
Standard deviation (STD) of the NN interval (SDNN)	*HRV*	*0.0903*	*4.86*	*1.36*	*No*
STD of differences between adjacent NN intervals (SDSD)	*HRV*	*-0.1232*	*2.84*	*0.85*	*No*
Number of interval differences of successive RR-intervals greater than 50 ms (NNI50)	*HRV*	*NA*	*0.00*	*100.00*	*Yes*
NNI50 divided by the number of NN intervals (PNNI50)	*HRV*	*NA*	*0.00*	*100.00*	*No*
Number of interval differences of successive RR-intervals greater than 20 ms (NNI20)	*HRV*	*NA*	*0.00*	*500.00*	*Yes*
NNI20 divided by the number of NN intervals (PNNI20)	*HRV*	*NA*	*0.00*	*100.00*	*No*
Root mean square of differences between adjacent NN intervals (RMSSD)	*HRV*	*-0.1232*	*2.84*	*0.85*	*No*
Median NN interval	*HRV*	*0.5021*	*57.09*	*8.62*	*No*
Difference between the maximum and minimum NN_interval	*HRV*	*0.2265*	*13.25*	*4.48*	*No*
RMSSD divided by the Mean NN interval	*HRV*	*-0.1856*	*-4.50*	*2.31*	*No*
SDSD divided by the Mean NN interval	*HRV*	*0.0735*	*-2.46*	*0.76*	*No*
Average heart-rate	*HRV*	*-0.5073*	*1.74*	*0.03*	*No*
Maximum heart-rate	*HRV*	*-0.8045*	*1.21*	*0.01*	*No*
Minimum heart-rate	*HRV*	*0.3449*	*8.61*	*1.31*	*No*
Heart-rate Std	*HRV*	*0.0496*	*1.71*	*1.19*	*No*
Low frequency power of the NN interval signal (LF)	*HRV*	*0.0414*	*6.86*	*2.70*	*No*
High frequency power of the NN interval signal (HF)	*HRV*	*-0.0263*	*4.89*	*2.27*	*No*
Ratio between LF and HF	*HRV*	*0.0101*	*0.68*	*1.44*	*No*
Normalized LF power	*HRV*	*1.4094*	*261.53*	*170.16*	*No*
Normalized HF power	*HRV*	*0.4899*	*9.39*	*5.18*	*No*
Total signal power	*HRV*	*0.0238*	*7.81*	*2.25*	*No*
Very low frequency power of the NN interval signal (VLF)	*HRV*	*0.0773*	*8.01*	*2.80*	*No*
Triangular_index of the NN-intervals distribution	*HRV*	*0.0638*	*2.32*	*0.86*	*No*
SD1 of the Poincaré plot	*HRV*	*-0.1232*	*2.61*	*0.88*	*No*
SD2 of the Poincaré plot	*HRV*	*0.1548*	*6.08*	*1.80*	*No*
Ration between SD2 and SD1	*HRV*	*-0.0636*	*1.00*	*0.89*	*No*
Cardiac Sympathetic Index (CSI)	*HRV*	*-0.0636*	*1.00*	*0.89*	*No*
Cardiac VagalIndex (CVI)	*HRV*	*0.8038*	*2.87*	*0.66*	*No*
Modified_CSI[1]	*HRV*	*0.0456*	*7.82*	*1.70*	*No*
2nd degree atrioventricular (AV) block burden	*Rhythm*	*NA*	*0.00*	*0.38*	*Yes*
3rd degree atrioventricular (AV) block burden	*Rhythm*	*NA*	*0.00*	*0.41*	*Yes*
Bigeminy rhythm burden	*Rhythm*	*NA*	*0.00*	*0.13*	*Yes*
Ectopic atrial rhythm burden	*Rhythm*	*NA*	*0.00*	*0.05*	*Yes*
Idioventricular rhythm burden	*Rhythm*	*NA*	*0.00*	*0.04*	*Yes*
Junctional rhythm burden	*Rhythm*	*NA*	*0.00*	*0.13*	*Yes*
Sinus block (pause) burden	*Rhythm*	*NA*	*0.00*	*0.02*	*Yes*
Supraventricular tachycardia (SVT) burden	*Rhythm*	*NA*	*0.00*	*0.04*	*Yes*
Normal sinus rhytm burden	*Rhythm*	*NA*	*1.00*	*-1.00*	*No*
Trigeminy rhythm burden	*Rhythm*	*NA*	*0.00*	*0.12*	*Yes*
Ventricular tachycardia (VT) burden	*Rhythm*	*NA*	*0.00*	*0.02*	*Yes*
Wenckebach rhythm burden	*Rhythm*	*NA*	*0.00*	*0.32*	*Yes*
Count of ectopic PAC	*Ectopic*	*NA*	*0.00*	*21.40*	*Yes*
Count of ectopic PVC	*Ectopic*	*NA*	*0.00*	*19.95*	*Yes*
Ectopic PAC burden	*Ectopic*	*NA*	*0.00*	*0.03*	*Yes*
Ectopic PVC burden	*Ectopic*	*NA*	*0.00*	*0.03*	*Yes*

Supplementary Table 1 – Manually extracted features and their normalization values.

The full set of features included in the model, along with their category, are listed in column Feature *and* Category. *The parameters used during the normalization step are reported in columns* Box-Cox Lambda *(*NA *if not applied), which refers to the single parameter of a standard Box-Cox transformation;* Offset*, which is subtracted from the feature value;* Scale*, which is the scaling divisor; and* 1 – exp(-x), *which is either* Yes *if the function is applied or* No *otherwise.*

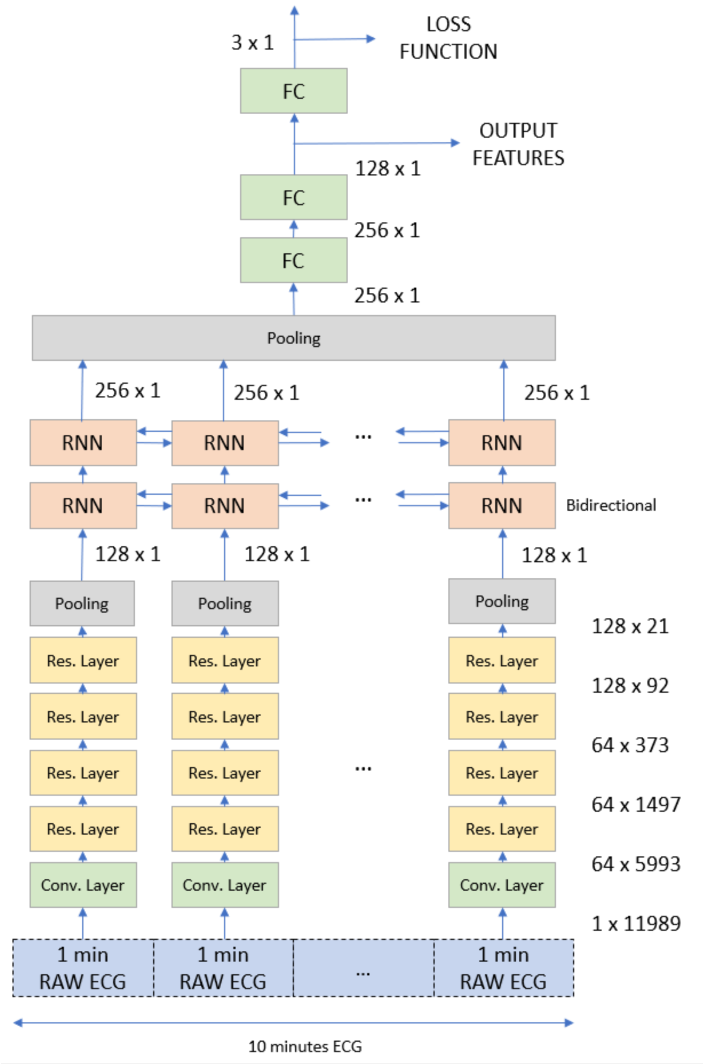

Supplementary Figure 3 – Detailed architecture of the DL Module A.

DL Module A is composed of two main steps. First, the 10 minutes ECG input is further divided into 1-minute smaller intervals. Then, a residual network processes each of these signals, and a bidirectional recurrent neural network (RNN) analyzes the temporal interactions. The outcome is processed with a fully connected network, and the features of DL module A are represented by the second last layer of the network. A single sample shape size is reported at the output of each block.

SUPPLEMENTARY REFERENCES

1. Jeppesen, J., Beniczky, S., Johansen, P., Sidenius, P. & Fuglsang-Frederiksen, A. Using Lorenz plot and Cardiac Sympathetic Index of heart rate variability for detecting seizures for patients with epilepsy. Annu Int Conf IEEE Eng Med Biol Soc **2014**, 4563-4566 (2014).
