# Supplementary material for: Prediction of atrial fibrillation from at-home single-lead ECG signals without arrhythmias
Source: NPJ Digit Med. 2023 Dec 12;6:229. doi: 10.1038/s41746-023-00966-w (PMC10716265; doi:10.1038/s41746-023-00966-w)
Supplement: Supplementary file 2 — Reporting Summary [file 41746_2023_966_MOESM2_ESM.pdf]

nature portfolio

Corresponding author(s): Giorgio Quer

Last updated by author(s): May 5, 2023

Reporting Summary

Nature Portfolio wishes to improve the reproducibility of the work that we publish. This form provides structure for consistency and transparency in reporting. For further information on Nature Portfolio policies, see our Editorial Policies and the Editorial Policy Checklist.

Statistics

For all statistical analyses, confirm that the following items are present in the figure legend, table legend, main text, or Methods section.

n/a	Confirmed	
☐	☒	The exact sample size (n) for each experimental group/condition, given as a discrete number and unit of measurement
☐	☒	A statement on whether measurements were taken from distinct samples or whether the same sample was measured repeatedly
☐	☒	The statistical test(s) used AND whether they are one- or two-sided<br>*Only common tests should be described solely by name; describe more complex techniques in the Methods section.*
☐	☒	A description of all covariates tested
☐	☒	A description of any assumptions or corrections, such as tests of normality and adjustment for multiple comparisons
☐	☒	A full description of the statistical parameters including central tendency (e.g. means) or other basic estimates (e.g. regression coefficient) AND variation (e.g. standard deviation) or associated estimates of uncertainty (e.g. confidence intervals)
☐	☒	For null hypothesis testing, the test statistic (e.g. F, t, r) with confidence intervals, effect sizes, degrees of freedom and P value noted<br>*Give P values as exact values whenever suitable.*
☒	☐	For Bayesian analysis, information on the choice of priors and Markov chain Monte Carlo settings
☐	☒	For hierarchical and complex designs, identification of the appropriate level for tests and full reporting of outcomes
☒	☐	Estimates of effect sizes (e.g. Cohen's d, Pearson's r), indicating how they were calculated

Our web collection on statistics for biologists contains articles on many of the points above.

Software and code

Policy information about availability of computer code

Data collection	The individuals included in this study were prescribed the iRhythm Zio XT patch (iRhythm Technologies Inc, San Francisco, CA) as part of routine care to monitor their heart rhythm for up to 14 days. The dataset used for the study was collected retrospectively.
Data analysis	Python 3.9 was utilized for code development. The deep learning models were developed and trained using PyTorch 1.11 and PyTorch-Lightning 1.6. For data manipulation and processing, the following libraries were employed: NumPy 1.21.5, pandas 1.4.1, Dask 2022.5.2, and SciPy 1.8.0. Visualization tasks were performed using seaborn 0.11.2 and Matplotlib 3.5.1. Data loading was facilitated by s3fs 2022.5.0, and parallelization of computation was achieved with Joblib 1.1.0.

For manuscripts utilizing custom algorithms or software that are central to the research but not yet described in published literature, software must be made available to editors and reviewers. We strongly encourage code deposition in a community repository (e.g. GitHub). See the Nature Portfolio guidelines for submitting code & software for further information.

Data

Policy information about availability of data

All manuscripts must include a data availability statement. This statement should provide the following information, where applicable:

- Accession codes, unique identifiers, or web links for publicly available datasets
- A description of any restrictions on data availability
- For clinical datasets or third party data, please ensure that the statement adheres to our policy

This is a retrospective study on an existing dataset. The data used in this study were obtained from iRhythm Technologies and are subject to restrictions that prevent public sharing. Detailed information regarding the data source and acquisition process is provided in the manuscript. Due to the terms of the data use agreement and compliance with ethical and legal requirements, the data cannot be made openly available.

Research involving human participants, their data, or biological material

Policy information about studies with human participants or human data. See also policy information about sex, gender (identity/presentation), and sexual orientation and race, ethnicity and racism.

Reporting on sex and gender	In our study, gender information was made available for each anonymized data sample, provided by iRhythm Technologies. We conducted gender-based analyses.
Reporting on race, ethnicity, or other socially relevant groupings	In our study, information about race was not available for the participants. However, our results are based on a test cohort that follows a natural distribution, which is assumed to be representative of the broader population.
Population characteristics	See section "Behavioural & social sciences study design" below
Recruitment	We included recordings acquired from January 2019 to May 2022, excluding individuals with persistent or near-persistent AF (defined as AF burden over 70%).
Ethics oversight	The protocol for this study was reviewed by the Scripps Office for the Protection of Research Subjects and determined to be exempt from formal committee review (IRB 21-7719).

Note that full information on the approval of the study protocol must also be provided in the manuscript.

Field-specific reporting

Please select the one below that is the best fit for your research. If you are not sure, read the appropriate sections before making your selection.

☒ Life sciences ☐ Behavioural & social sciences ☐ Ecological, evolutionary & environmental sciences

For a reference copy of the document with all sections, see nature.com/documents/nr-reporting-summary-flat.pdf

Life sciences study design

All studies must disclose on these points even when the disclosure is negative.

Sample size	A total of 459,889 recordings, obtained from 446,900 unique individuals, were analyzed in this study.
Data exclusions	Individuals with persistent or near-persistent AF (defined as AF burden over 70%) were excluded from the study.
Replication	All the proposed metrics in our study are deterministic, ensuring that the results are fully reproducible. The entire processing pipeline has been thoroughly tested, consistently yielding the same results from the raw data to the final outcomes.
Randomization	Participants were randomly allocated to three distinct cohorts: training, calibration, and testing. We took measures to ensure that individuals in the testing cohort were not present in the training or calibration cohorts, thus maintaining the independence of the samples. The detailed procedure for this allocation process can be found in the manuscript.
Blinding	The random group allocation in our study was carried out by a computer program, ensuring that the process was blinded to the investigators.

Reporting for specific materials, systems and methods

We require information from authors about some types of materials, experimental systems and methods used in many studies. Here, indicate whether each material, system or method listed is relevant to your study. If you are not sure if a list item applies to your research, read the appropriate section before selecting a response.

Materials & experimental systems

n/a	Involved in the study
☒	☐ Antibodies
☒	☐ Eukaryotic cell lines
☒	☐ Palaeontology and archaeology
☒	☐ Animals and other organisms
☒	☐ Clinical data
☒	☐ Dual use research of concern
☒	☐ Plants

Methods

n/a	Involved in the study
☒	☐ ChIP-seq
☒	☐ Flow cytometry
☒	☐ MRI-based neuroimaging
